# Turning Uridines around: Role of rRNA Pseudouridylation in Ribosome Biogenesis and Ribosomal Function

**DOI:** 10.3390/biom8020038

**Published:** 2018-06-05

**Authors:** Marianna Penzo, Lorenzo Montanaro

**Affiliations:** Department of Experimental, Diagnostic and Specialty Medicine, Alma Mater Studiorum University of Bologna, Via Massarenti 9, 40138 Bologna, Italy

**Keywords:** pseudouridylation, rRNA, ribosome biogenesis, X-linked dyskeratosis congenita, cancer, mRNA translation, ribosome diversity, translational control, internal ribosome entry site-mediated translation

## Abstract

Ribosomal RNA (rRNA) is extensively edited through base methylation and acetylation, 2′-O-ribose methylation and uridine isomerization. In human rRNA, 95 uridines are predicted to by modified to pseudouridine by ribonucleoprotein complexes sharing four core proteins and differing for a RNA sequence guiding the complex to specific residues to be modified. Most pseudouridylation sites are placed within functionally important ribosomal domains and can influence ribosomal functional features. Information obtained so far only partially explained the degree of regulation and the consequences of pseudouridylation on ribosomal structure and function in different physiological and pathological conditions. This short review focuses on the available evidence in this topic, highlighting open questions in the field and perspectives that the development of emerging techniques is offering.

## 1. RNA Pseudouridylation and Its Roles in Ribosome Biogenesis

**Figure 1:**
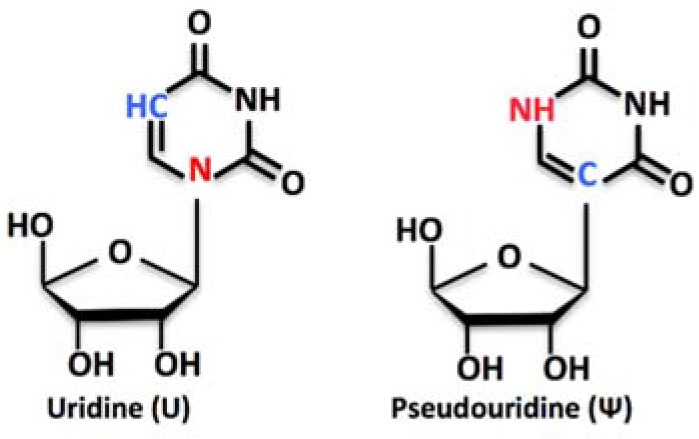
Structure formulae of uridine and pseudouridine.

Pseudouridine (Ψ) is the 5-ribosyl isomer of uridine (Figure 1). It derives from the uracil base rotation of 180°, which makes the uracil attached to the 1′ carbon (C1´) of the ribose via a carbon-carbon instead of a nitrogen-carbon glycosidic bond (see [1,2] for a broader review). Pseudouridine (Ψ) is the most prevalent within more than 100 different modified nucleosides found in RNA, with about 9500 Ψ residues identified in mammals and yeast and deposited in the RMBase database [3]. Pseudouridine (Ψ) is found in all species and in many classes of RNA, including ribosomal RNA (rRNA), transfer RNA (tRNA), mitochondrial tRNAs (Mt-tRNAs), small Cajal Body-specific RNAs (scaRNAs), small nucleolar RNAs (snoRNAs), microRNAs (miRNAs), long intergenic non-coding RNAs (lincRNAs), messenger RNAs (mRNAs), and other miscellaneous RNAs (misc_RNAs) [4]. Uridine isomerization to Ψ is carried out by either RNA-driven enzymatic complexes or stand-alone enzymes, depending on the class of the RNA harboring the target residue. In rRNA, Ψs are generated by RNA-guided enzymatic complexes, and they account for about the 1.4% of all bases, with a total of 95 predicted Ψs in human 28S, 18S, 5.8S, and 5S rRNAs [5,6,7]. In bacteria and yeast rRNAs, the number of Ψs is significantly lower (36 in *E. coli*, 46 in *S. cerevisiae* [8]), mirroring the complexity level of these organisms.

The presence of an extra hydrogen bond donor at its non-Watson-Crick edge endows Ψ with biochemical and biophysical properties distinct from those of uridine and all other known nucleotides. In particular, the presence of Ψ is able to provide: (i) greater rigidity to the phosphodiester backbone of the RNA; (ii) stabilization of Ψ-A base pairs (compared to that of U-A base pairs) through some effects on base stacking and water coordination, thus affecting RNA structure, spatial conformation and, ultimately, its functional properties; and (iii) increased thermal stability (reviewed in [9]).

The site-specific modification of target uridines in rRNA, as for other modification types occurring in this class of RNA, represents an extremely important passage of ribosome synthesis. Many aspects of uridine modification process are now quite clear, including the players involved, the timing and the cellular localization of the processes, and the position on rRNAs in the three-dimensional structure of the ribosome (even though all of this information might not be available for each single modification) [10]. Still, to understand the function (or functions) of many of these Ψs remains a demanding challenge for investigators in the field.

Pseudouridylation, together with other rRNA modifications, is only one aspect of the process of preparation of rRNAs for ribosome biogenesis. As discussed later on, some of these modifications are considered important for ribosome production to occur, since this process is characterized by the presence of multiple control steps, which, in the end, ensure the production of competent ribosomes. 

Ribosomal RNA is synthesized by two different RNA polymerases. While 5S rRNA is transcribed by RNA PolIII in the nucleoplasm, mature 5.8S, 18S, and 28S rRNAs are derived from a unique precursor, termed 47S, also harboring two external transcribed spacers (5′ and 3′ external transcribed spacer (ETS)) and two internal transcribed spacers (ITS1 and 2) transcribed in the nucleolus by RNA PolI. ETS and ITS sequences are sequentially removed by exo- and endo-nucleolytic cleavages, in coordinated series of processing events that may present variations depending on cellular type or status (for a broader review of the topic please refer to [11]). In rRNA, pseudouridylation is carried out by ribonucleoprotein (RNP) complexes called H/ACA box RNPs, each consisting of one H/ACA snoRNA and four core proteins, namely GAR1, NHP2, NOP10 and dyskerin (DKC1). H/ACA snoRNAs contain a conserved ANANNA sequence called “hinge box” (indicated with H) and a sequence of three nucleotides (ACA) present at their 3′ end (called ACA box). Structurally H/ACA snoRNAs are characterized by the presence of a double hairpin, each harboring a pseudouridylation pocket, which is specific to a particular target sequence, based on sequence-specific base pairing. The interaction between the guide snoRNA and the substrate guides the enzymatic complex on the target uridine. A further stabilization occurs thanks to additional interactions of the substrate RNA with protein component of small nucleolar ribonucleoproteins (snoRNPs) and in particular with DKC1, which carries out the isomerization of the target uridine (see [5,12] for a comprehensive review).

Most interactions between pre-rRNA and pseudouridylation RNP complexes may occur co-transcriptionally; indeed in yeast it has been shown that pre- rRNA transcripts are first completed and modified before further processing, probably because modifications are required for the acquisition of structural conformations necessary for the occurrence of later processing events [13]. On this regard, it is not definitively clear if each of the predicted snoRNA guided associations also require the modification to occur or if instead, in some cases, the molecular interaction is sufficient to allow for the processing. 

## 2. Role of Pseudouridine Formation on Ribosome Function

Considering the three dimensional (3D) structure of the mature ribosome, it is also interesting to notice that many pseudouridylation sites are placed in (or very near to) functionally important ribosomal sites, such as tRNA and mRNA binding sites [14]. This suggests that pseudouridylation at these specific sites could impact on ribosomal function. Interestingly, while pseudouridylation is required for ribosome biogenesis and ribosomal function in eukaryotes, it has been possible to generate strains of bacteria lacking completely rRNA pseudouridylation and displaying only minor defects of ribosome biogenesis and ribosomal function [15]. These findings suggest a differential requirement for Ψ modifications for prokaryotic and eukaryotic cells. Recent studies aimed to study the role of specific Ψs in functionally important sites in yeast ribosomes, by deleting snoRNA sequences guiding these specific modifications. By this approach, it. was shown that depleting yeast cells of snoRNAs guiding modifications in the peptidyl transferase center alters ribosome function particularly when a conserved Ψ modification in the A site of tRNA binding is lost [16]. In addition, loss of Ψ residues at the intersubunit bridge, with particular reference to the helix 69 in the large ribosomal subunit, may induce a number of structural and functional effects including reduced amino acid incorporation, increased stop codon readthrough, and increased sensitivity to antibiotics targeting the ribosome [17]. Moreover, loss of modification in the decoding center has complex, position-dependent, outcomes that span from no apparent effects (for single modification loss) to reduced amino acid incorporation and delayed cell growth, to strong ribosome biogenesis defects [18].

On the other hand, a greatly lower amount of data is available in human/mammalian systems, and the information we have is mostly linked to human disorders such as inherited disorders involving genes encoding for components of the pseudouridylation complex and cancer and is therefore discussed in the following section.

## 3. rRNA Pseudouridylation and Disease

The first evidence linking rRNA pseudouridylation and human disease dates back to 20 years ago, when mutations of the *DKC1* gene encoding the pseudouridine synthase dyskerin were identified in X-linked dyskeratosis congenita (X-DC) patients [19]. X-DC is a multisystemic syndrome characterized by failure of proliferating tissue (skin, mucosae and bone marrow) and increased cancer susceptibility [20]. As reported above dyskerin is part of the pseudouridylation complex mediating pseudouridylation in rRNA and therefore X-DC has been defined as a ribosomopathy: a disease due to ribosome malfunction. Soon afterwards dyskerin was found to be part of the telomerase complex through direct interaction with telomerase RNA component (TERC) [21] and the pathogenesis of X-DC was therefore ascribed also to telomere attrition [22]. To better dissect this issue, studies in *DKC1* hypomorphic mouse models were conducted. These showed that a recapitulation of the clinical defects observed in X-DC patients, including cancer susceptibility and bone marrow failure, takes place together with a reduction of the global levels of Ψs in rRNA and with an impairment in the translation of a group of cellular mRNAs [23,24]. These mRNAs contain specific sequence elements termed internal ribosome entry site (IRES) [23]. IRES elements are highly structured nucleotide sequences, originally reported in viral mRNAs but present also in a limited number of eukaryotic cellular mRNAs [25], that can mediate translation initiation independently of the canonical cap-dependent mechanism. The experimentally verified translational targets found in cells expressing low dyskerin levels include Bcl-xL and XIAP (X-linked inhibitor of apoptosis protein) mRNAs, encoding for antiapoptotic factors [23], and p27(Kip1) and p53 [23,26,27] mRNAs, encoding for tumor suppressors. In addition, the translation of other IRES-containing mRNAs (such as vascular endothelial growth factor -VEGF and heat shock protein 70-HSP70 mRNAs [28]) increase after DKC1 depletion, indicating a complex remodulation of translational regulation in these conditions. Likewise, the translational fidelity is also strongly lowered in cells after partial DKC1 depletion [29]. Importantly, experiments performed by means of a reconstituted cell-free in vitro translation system ascribed all the observed translational changes to the intrinsic ribosomal alterations induced by reduced global pseudouridylation of rRNA [30]. These observations, highlighting the effects of rRNA pseudouridylation reduction on translational control, may help explaining both the defect in proliferating tissues and the cancer susceptibility typical of X-DC [31]. Interestingly, mutations of the *DKC1* gene have been also reported in familial interstitial pneumonia [32]. 

In addition to these inherited disorders, a subset of human cancers arising in the general population display reduced dyskerin expression and functions and low rRNA pseudouridylation suggesting that the observed translational defects may also contribute to the neoplastic phenotype in a fraction of sporadic tumors [31,33]. On this concern, a highly variable dyskerin expression has been observed in a variety of human tumor types, including breast, lung, prostate, colon, head and neck, and hepatocellular carcinomas [31,34,35,36]. In general, low dyskerin expression is associated with a favorable clinical behavior [35,36,37].

To appreciate the contribution of altered rRNA pseudouridylation in inherited disorders and in tumorigenesis, the expression of components of H/ACA snoRNP complexes in different pathological conditions should be considered beyond altered DKC1 expression. Autosomal forms of Dyskeratosis Congenita can derive from mutations of *NOLA2* or *NOLA3* encoding for NHP2 and NOP10, respectively [38,39]. Similarly to dyskerin, the expression of these genes is also found altered in human neoplasms and is associated with disease aggressiveness [40]. As for *DKC1*, the products of *NOLA2* and *NOLA3* genes are both involved in telomerase function and in RNA pseudouridylation. 

In addition, the expression of snoRNAs has been found to be deregulated in a series of human disorders including cancer, neurodegenerative, and viral diseases and also in response to stress and treatment with specific drugs [41]. The expression of different H/ACA snoRNAs is deregulated in a series of human neoplasms of different origins (see McMahon et al. for review [42]). On this regard, it is important to consider that snoRNA expression is under the control of tumor suppressive and oncogenic signaling pathways [43,44], which can therefore alter rRNA modification levels during the cellular transformation process. Of note, the majority of these studies analyzed snoRNAs expression from library preparations specific for small RNAs, implying the loss of a large fraction of snoRNA of middle to large size. The frequency of these alterations could be therefore largely underestimated. In addition, recent genome wide studies identified frequent alterations in snoRNA genes in human cancer, including in particular gene amplification and homozygous deletion [45,46,47]. An implication of these findings is that in particular pathological contexts rRNA pseudouridylation can be altered by the relative lack or abundance of the activity of factors mediating the modification.

Very recently, high-resolution structural cryogenic electron microscopy (cryo-EM) studies demonstrated that, in human ribosomes, rRNA modifications are distributed into a wide ribosome portion including important functional domains. There is evidence that such modifications are altered in cancer cells [48]. Such studies are currently in a pioneering stage and it can be expected that provided that the required methodology will become available a large amount of information regarding rRNA pseudouridine modification in cancer could be obtained, shedding light on its relevance in ribosomal function and cancer development.

**Figure 2:**
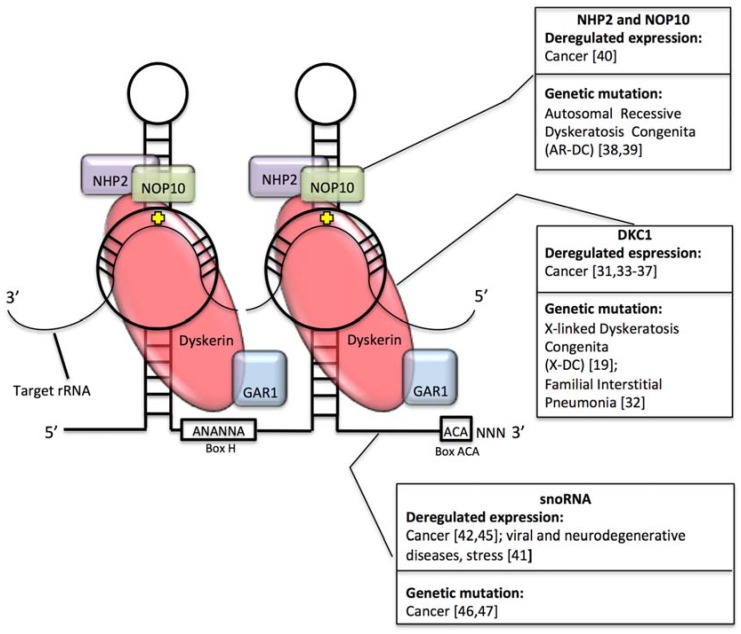
Schematic representation of the Box H/ACA riboucleoprotein (RNP) pseudouridylation complex, with particular reference to the diseases associated to alterations in the expression of each component.

Figure 2 outlines how defects in the H/ACA RNP pseudouridylation complex may be involved in different human disorders.

## 4. Are Ribosome All Modified at the Same Manner?

Ribosomal RNA modifications are thought to be one of the main sources of ribosomes heterogeneity, either in physiological or in pathological conditions. Intriguingly, different studies have recently clarified that subpopulations of diversely modified mature ribosomes may co-exist in single cells ([49,50,51]. In these studies next-generation sequencing-based RiboMethSeq [52] mapping of 2′-O-methylated rRNA residues highlighted the existence of sub-stoichiometrically modified sites, implying that not all rRNAs are similarly modified. It is worthwhile to underline that such an approach can be very informative for 2′-O-methylation, which is by itself exploitable for direct and confident identification by high-throughput sequencing techniques. Unfortunately, the equivalent high-throughput approaches for Ψ detection (Pseudo-Seq, Ψ-Seq, PSI-Seq [53,54,55]) should be interpreted with caution, since they are all based on the use of *N*-cyclohexyl-*N*’-β-(4-methylmorpholinium)ethylcarbodiimide (CMC), which has been reported to bind with inconstant efficiency, thus possibly introducing a bias for quantitative interpretation of results [56]. The proof of the existence of diversified ribosomes pools is, in line of principle, a very strong indication of ribosome functional specialization. Indeed, functional assays performed in different cellular backgrounds have already proven that pseudouridylation defects may alter translational competence [28,29,30,33], and we have shown that intrinsic ribosomal functional defects arise as a consequence of rRNA pseudouridylation down-regulation [30]. However, technical limitations have so far impeded the study of ribosome diversity with the needed sensitivity. Initial studies conducted to identify pseudouridylation sites on rRNA were based on the use of CMC in conjunction with Sanger-based sequencing techniques [57]. More recently, in the era of next generation sequencing (NGS), several different approaches have been developed to identify and quantify Ψs at specific positions in RNAs [53,54,55,58]. Albeit these techniques are highly sensitive, in addition to what above reported, they have been applied to the research of pseudouridylated sites in whole cellular RNAs and therefore did not fully address the issue of the abundance of specific Ψ residues on mature ribosomes. Moreover, the high amount of rRNA may interfere in the achievement of an adequate coverage by RNAseq in order to obtain a quantitative evaluation of the number of modified sites. In addition to sequencing techniques, cryo-EM was, as mentioned above, recently exploited to localize and visualize rRNA modifications directly in mature, 80S ribosomes from HeLa cells [48]. Thanks to an extremely high resolution (up to 2.5 Å), it was possible to address the 3D localization of many modified residues in rRNAs, even though only a small fraction of the predicted modifications was identified on mature ribosomes due to technical limitations. For instance, only 21 Ψs were confirmed by this technique out of the expected 95. The 95 predicted Ψs have never been identified before on mature ribosomes; instead, they were either predicted based on sequence-specificity of guide snoRNAs or mapped by sequencing approaches on total cellular rRNAs, and therefore could not faithfully mirror the modification status of each single residue in mature ribosomes. (see https://www-snorna.biotoul.fr/index.php, [8,59]). These considerations also open up new questions about the inducible nature of rRNA modifications in general, and of pseudouridylation in particular, paralleling what has been proposed for 2′-O-methylation, that is the presence of a core of conserved modifications sites, associated to a peripheral shell of “vulnerable” sites being modified in a regulated manner in different settings [50]. Indeed, it is possible that only a fraction of the predicted Ψs are necessary for a correct rRNA maturation/assembly with ribosomal proteins, and the rest may be added only in specific cellular contexts. In addition, it is possible that rRNA modifications in human ribosomes may differ between cell types, or within the same cell type, between physiology and pathology. Studies addressing these issues are probably slowed down by the lack of appropriate technical approaches, allowing for physical particle sorting and analysis needed for the identification of structurally/functionally distinguishable ribosomal (sub)populations. 

From a functional standpoint, the possibility to assay the activity of diversely modified ribosomes might be even more challenging. We have set up a cell free translation system suitable for testing, in a controlled setting, different aspects of the translational activity of ribosomes stringently purified from cultured cells [60]. Even though this tool is extremely helpful to investigate the functional outcomes of structural alterations in widely represented ribosomal populations within cells, it does not allow evaluating the translational contribution of ribosomal sub-populations that are less abundant. To this end, innovative approaches are needed, aiming to separate ribosomal subpopulations on a structural basis prior to their functional testing.

## 5. Concluding Remarks

The importance of rRNA pseudouridylation and of its regulation (as for other modifications) has been largely underexplored for many reasons. The impact of the presence/absence of a single modification or combinations of a limited number of modifications has been explored only in yeast, and the scientific community currently has limited information on how this could be important in regulating mammalian ribosome function, protein synthesis, and gene expression in different physiological states and in many human disorders. For instance, it is known that under mammalian target of rapamycin (mTOR) activation, specific residues can be modified upon up-regulation of single H/ACA guide RNA [61], and something similar could happen under different stimuli.

In addition, the timing of specific modifications may be under some kind of control in mature ribosomes, since many sites lie on exposed domains potentially reachable by the pseudouridylation complexes, similarly to what has been reported for mRNA [62].

Moreover, at the specific site level, the regulation of the modification may depend on the availability of guide RNAs or on the accessibility/masking of the substrate by means of additional factors. To clarify these points may represent an important area of research for cellular and molecular biologists, with the potential to provide unconsidered keys for interpreting gene expression regulation in many pathological processes. Recent results suggest that it may not be rigorous enough to assume the presence or the absence of a single modification when assaying it using only one kind of technical method.

To obtain a complete picture, therefore, will require the development of multiple additional model systems and further technical approaches both at the structural and functional level and the development of a solid awareness regarding ribosome-mediated gene expression control, a scientific challenge we are currently only begin to tackle.
